# Live probiotic bacteria administered in a pathomimetic Leaky Gut Chip ameliorate impaired epithelial barrier and mucosal inflammation

**DOI:** 10.1038/s41598-022-27300-w

**Published:** 2022-12-31

**Authors:** Soyoun Min, Nam Than, Yong Cheol Shin, Grace Hu, Woojung Shin, Yoko M. Ambrosini, Hyun Jung Kim

**Affiliations:** 1 Department of Inflammation and Immunity, Lerner Research Institute, Cleveland Clinic, 9500 Euclid Ave., NE3, Cleveland, OH 44195 USA; 2 Department of Biomedical Engineering, The University of Texas at Austin, Austin, TX 78712 USA; 3 Wyss Institute for Biologically Inspired Engineering, Harvard University, Boston, MA 02115 USA; 4 Institute for Medical Engineering and Science, Massachusetts Institute of Technology, Cambridge, MA 02139 USA; 5 Department of Veterinary Clinical Sciences, College of Veterinary Medicine, Washington State University, Pullman, WA 99164 USA

**Keywords:** Biomimetics, Regenerative medicine, Tissue engineering, Biomedical engineering, Gastrointestinal models, Microbiology

## Abstract

Here, we report a pathomimetic Leaky Gut Chip that recapitulates increased epithelial permeability and intestinal inflammation to assess probiotic intervention as live biotherapeutics. We leveraged a mechanodynamic human gut-on-a-chip (Gut Chip) that recreates three-dimensional epithelial layers in a controlled oxygen gradient and biomechanical cues, where the addition of a cocktail of pro-inflammatory cytokines, TNF-α and IL-1β, reproducibly induced impaired epithelial barrier followed by intestinal inflammation. This inflamed leaky epithelium was not recovered for up to 3 days, although the cytokine treatment ceased. However, when probiotic bacteria, either *Lactobacillus rhamnosus* GG or a multi-species mixture (VSL#3), were respectively administered on the leaky epithelium, bacterial cells colonized mucosal surface and significantly improved barrier function, enhanced the localization of tight junction proteins such as ZO-1 and occludin, and elevated mucus production. In addition, inflammatory markers, including p65, pSTAT3, and MYD88, that were highly expressed in the germ-free control were significantly reduced when probiotic bacteria were co-cultured in a Leaky Gut Chip. Probiotic treatment also significantly reduced the production of secretory pro-inflammatory cytokines. Hence, our pathomimetic Leaky Gut Chip may offer a translational strategy to dissect the therapeutic mechanism of live biotherapeutic products and validate their clinical potential by incorporating patient-derived organoids.

## Introduction

The “leaky gut” is an unspecified clinical condition with an abnormal elevation of epithelial permeability in the human intestine^1–3^. A leaky epithelial barrier often leads to the invasion of pathogenic bacteria or even the gut microbiota, uncontrolled transport of exogenous molecules or bacterial toxins into lamina propria and bloodstream, diffusion of oxygen into the lumen, and elevated immune responses^4^. Notably, a leaky gut may cause various health concerns in intestinal host and microbial cells, such as chronic inflammation and microbial dysbiosis^5–7^. Recent studies have revealed that a leaky barrier may be associated with autoimmune disorders (e.g., systemic lupus erythematosus)^8^, inflammatory bowel disease (e.g., Crohn’s disease and ulcerative colitis)^9^, and neurodegenerative diseases (e.g., autism spectrum disorder)^10^, suggesting that the management of an impaired epithelial barrier can be a necessary preventive intervention of complicated inter-organ diseases.

One of the promising approaches to mitigate leaky barrier function is introducing probiotic bacteria as an administration of live biotherapeutics^11,12^. Indeed, the effects of probiotic interventions have been validated in various in vivo animal models or human clinical studies, including improvement of intestinal barrier function, restoration of the diversity of gut microbiota, and amelioration of inflammatory responses^13–15^. Several preclinical studies also demonstrated that specific probiotic strains improved epithelial barrier dysfunction^16,17^ and remedied intestinal inflammation^18,19^. Hence, probiotic intervention can be considered a complementary and integrative strategy to control pathological leaky epithelial conditions.

However, there is ongoing controversy on the use of probiotics in terms of the consistency of clinical efficacy^20^ and the reproducibility of experimental outcomes in various surrogate models^21^. Notably, it has been challenging to experimentally investigate the beneficial effect of probiotic bacteria on the leaky gut, where limited models to validate their preventive and therapeutic functions have remained to be resolved. For instance, animal models are complicated to induce a leaky epithelial barrier to test a probiotic regimen exclusively. Furthermore, multi-cellular responses and complex immunological factors often complicate the understanding of the independent contribution of a specific probiotic strain on barrier restoration^22^. In addition, poor colonization of administered probiotic strains (e.g., *Escherichia coli* Nissle 1917^23^) hampers reliable experimental interpretation. In case of in vitro models, one of the crucial challenges to reducing the knowledge gap is a lack of human-relevant intestine models that enable the quantitative assessment of host responses to probiotic administration. Notably, static cell culture models make it difficult to perform longitudinal host–probiotics interactions to accurately dissect their complex crosstalk at the mucosal interface with cellular and molecular resolution^24,25^. Conventional cell cultures also do not provide in vivo-relevant cytodifferentiation and three-dimensional (3D) epithelial layers, which hinders accurate host–probiotic interactions.

Alternatively, we have developed a gut-on-a-chip (Gut Chip) microphysiological system that recapitulates intestinal epithelial barrier, physiodynamic mucosal microenvironment, cytodifferentiation and 3D histogenesis, physiological epithelial functions, and long-term host–microbiome co-cultures in a living human gut^24^. The Gut Chip can offer an accurate oxygen gradient in situ to permit the co-culture of obligate anaerobic gut bacteria or fecal microbiota with a wide range of host epithelium from immortalized human intestinal epithelial lines to patient-derived organoid epithelium^26,27^. Importantly, we dissected complex immune responses and inflammatory cascades in a modular way by independently coupling individual disease factors (i.e., epithelium, immune components, inflammatory triggers, bacterial toxin, and gut bacteria) and identified the underlying mechanism by which an intact epithelial barrier is necessary and sufficient to maintaining physiological tolerance^28^.

In this study, we leveraged a microphysiological Gut Chip to recapitulate pathological leaky gut symptoms by introducing potent pro-inflammatory cytokines under biomechanical stimulations and oxygen control. By administering two commercially available probiotics, either *Lactobacillus rhamnosus* GG (LGG) or a probiotic formulation that contains 8 different probiotic strains (VSL#3), respectively, beneficial functions of probiotic treatment associated with barrier restoration and anti-inflammatory responses were demonstrated in a Leaky Gut Chip. Finally, we discussed the potential directions of our proof-of-principle study toward advanced live biotherapeutic strategies to manage various gastrointestinal (GI) diseases.

## Results

### A microfluidic Leaky Gut Chip demonstrates an impaired epithelial barrier

**Figure 1:**
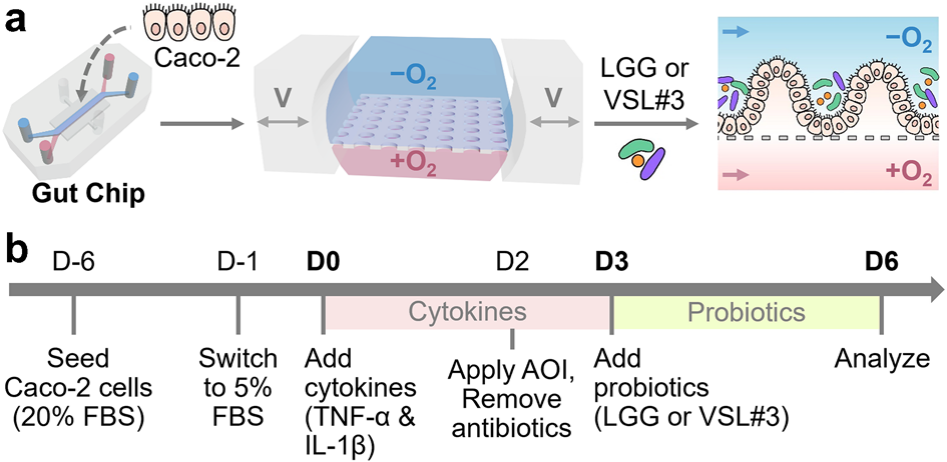
The experimental design of the creation of a Leaky Gut Chip for validating probiotic effects. (**a**) The workflow illustrating the growth of intestinal epithelial Caco-2 cells in a Gut Chip that employs a physiological oxygen gradient and host-probiotics co-cultures with either LGG or VSL#3 cells. The “−O_2_” and “+O_2_” mean the absence and the presence of oxygen in the culture medium, respectively. Bidirectional grey arrows in the middle schematic show cyclic mechanical deformations in a Gut Chip. V, cyclic vacuum suctions. A dashed line in the right schematic shows the location of a porous basement membrane in a Gut Chip. Blue and pink arrows display the direction of the culture medium in a Gut Chip. (**b**) An experimental timeline to induce a leaky epithelial barrier and cellular inflammation followed by probiotic amelioration. Epithelial Caco-2 cells are cultured in a Gut Chip under flow and motions for 5–6 days at a high serum condition (20% (v/v) FBS), then switched to a low-serum (5% FBS) culture medium that contains a cytokine cocktail (TNF-α and IL-1β) for 3 days. Probiotic co-cultures were performed after epithelial cells were stabilized to an oxygen gradient and an antibiotic-free condition. During the probiotic co-cultures, cytokine treatment was discontinued. AOI, anoxic–oxic interface; Abx, antibiotics. “D” indicates the day of cultures. We set D0 as the first day of cytokine treatment. D-1 and D-6 denote the pre-culture of Caco-2 cells in a Gut Chip on 1 and 6 days before the cytokine treatment, respectively. The pink and green boxes indicate the period of a cytokine challenge and a probiotic co-culture, respectively.

**Figure 2:**
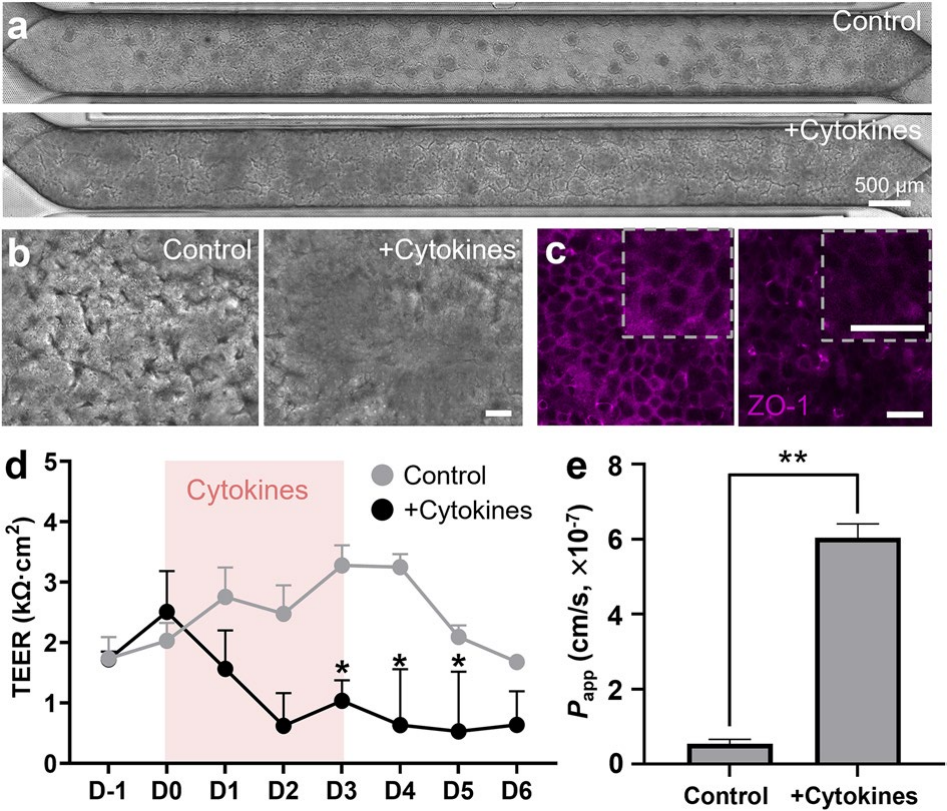
The recreation of a leaky epithelial layer in a Leaky Gut Chip. (**a**) The stitched phase-contrast views of epithelial layers across the entire microchannel in the absence (Control) or the presence of a cytokine cocktail (+ Cytokines; 100 ng/mL TNF-α and 100 ng/mL IL-1β) taken on day 3 (D3) since the cytokine treatment. (**b**) High-power magnification of epithelial morphology visualized by DIC microscopy in the absence (Control) or the presence of a cytokine cocktail (+ Cytokines). (**c**) Immunofluorescence micrographs that highlight ZO-1 in the absence (Control) or the presence of a cytokine cocktail (+ Cytokines). An inset (a dashed square) in each panel shows a high-power magnification of the part of the provided immunofluorescence image. (**d**) The profile of epithelial barrier function quantitated by TEER measurement as a function of time in the absence (Control; n = 5) or the presence of a cytokine cocktail (+ Cytokines; n = 6). A pink box in the chart illustrates the period of cytokine treatment for 72 h. *p < 0.05. (**e**) Apparent permeability (*P*_app_) calculated by the fluorescein transport assay across the epithelial interface in a Leaky Gut Chip without (Control) or with a cytokine mixture (+ Cytokines). **p < 0.01. Bars are 50 µm, unless otherwise indicated.

We leveraged a mechanodynamic Gut Chip that enables us to recreate three-dimensional (3D) layers of human intestinal Caco-2 epithelial cells on an extracellular matrix (ECM)-coated elastic porous membrane to recreate a Leaky Gut Chip (Fig. 1a)^24,29^. Under in vivo-relevant luminal flow and rhythmical cyclic deformations that mimic peristalsis-like biomechanical physiology, we manipulated this intestinal mucosal microenvironment to simulate leaky gut symptoms by adding a cocktail of pro-inflammatory cytokines that contained 100 ng/mL tumor necrosis factor (TNF)-α and 100 ng/mL interleukin (IL)-1β^30,31^. To induce epithelial barrier disruption followed by inflammatory responses, we pre-conditioned the 3D epithelial layers with reduced serum (from 20% v/v fetal bovine serum, FBS, to 5%) that can minimally elicit epithelial proliferation due to the high-serum condition. Under this condition, we exclusively investigated the contribution of probiotic intervention to the management of leaky epithelium. Next, we sequentially challenged epithelial cells with a cytokine cocktail for 3 days, ceased the cytokine treatment, then monitored how the cytokine challenge perturbs epithelial morphology and the expression of tight junction proteins known to be a hallmark of the leaky gut syndrome (Fig. 1b). The stitched (Fig. 2a, Control) and the zoomed-in micrographs (Fig. 2b, Control) showed a 3D epithelial protrusion with repeated inter-villus crevices reminiscent of in vivo intestinal villi, which is the landmark morphological characteristics of intestinal epithelium grown in a Gut Chip^24^. However, the treatment of pro-inflammatory cytokines substantially compromised the cell–cell junctions and 3D epithelial morphology (Fig. 2a,b, + Cytokines), where the undulated epithelial layers lost the epithelial boundary contour and showed a highly blurry apical surface with darkened cell layers compared to the cytokine-free control. The expression and localization of tight junction protein zonula occludens-1 (ZO-1) were also seriously disrupted under a 3-day-long cytokine treatment (Fig. 2c). The ZO-1 protein that was uniformly organized in the cytokine-free control was notably perturbed with a lack of uniform distribution (Supplementary Fig. S1). Furthermore, we verified that the cytokine treatment reproducibly induced decreased epithelial barrier integrity quantitated by transepithelial electrical resistance (TEER; Fig. 2d). The cytokine-free control showed a progressive increase of TEER during the entire culture period, followed by a spontaneous reduction of barrier function over time (from D4 in Fig. 2d, Control) as previously observed^24^. On the contrary, adding a cytokine cocktail significantly decreased the TEER value even after the cessation of cytokine treatment (Fig. 2d, + Cytokines), suggesting that Caco-2 epithelium failed to restore barrier integrity showing a leaky gut symptom for at least 6 days since the cytokine challenge was applied. The apparent permeability coefficient (*P*_app_) using fluorescein (376.27 Da) on 3D epithelial layers also quantitatively supported the barrier dysfunction (Fig. 2e), where the apparent permeability increased significantly (*p* < 0.01) in the cytokine-treated group (6.04 ± 0.53 × 10^−7^ cm/s) compared to the control (0.54 ± 0.17 × 10^−7^ cm/s), supporting that the pathological hyper-permeability of the leaky gut syndrome was successfully recapitulated in our microengineered Leaky Gut Chip.

### A Leaky Gut Chip illustrates longitudinal host–probiotic co-cultures

**Figure 3:**
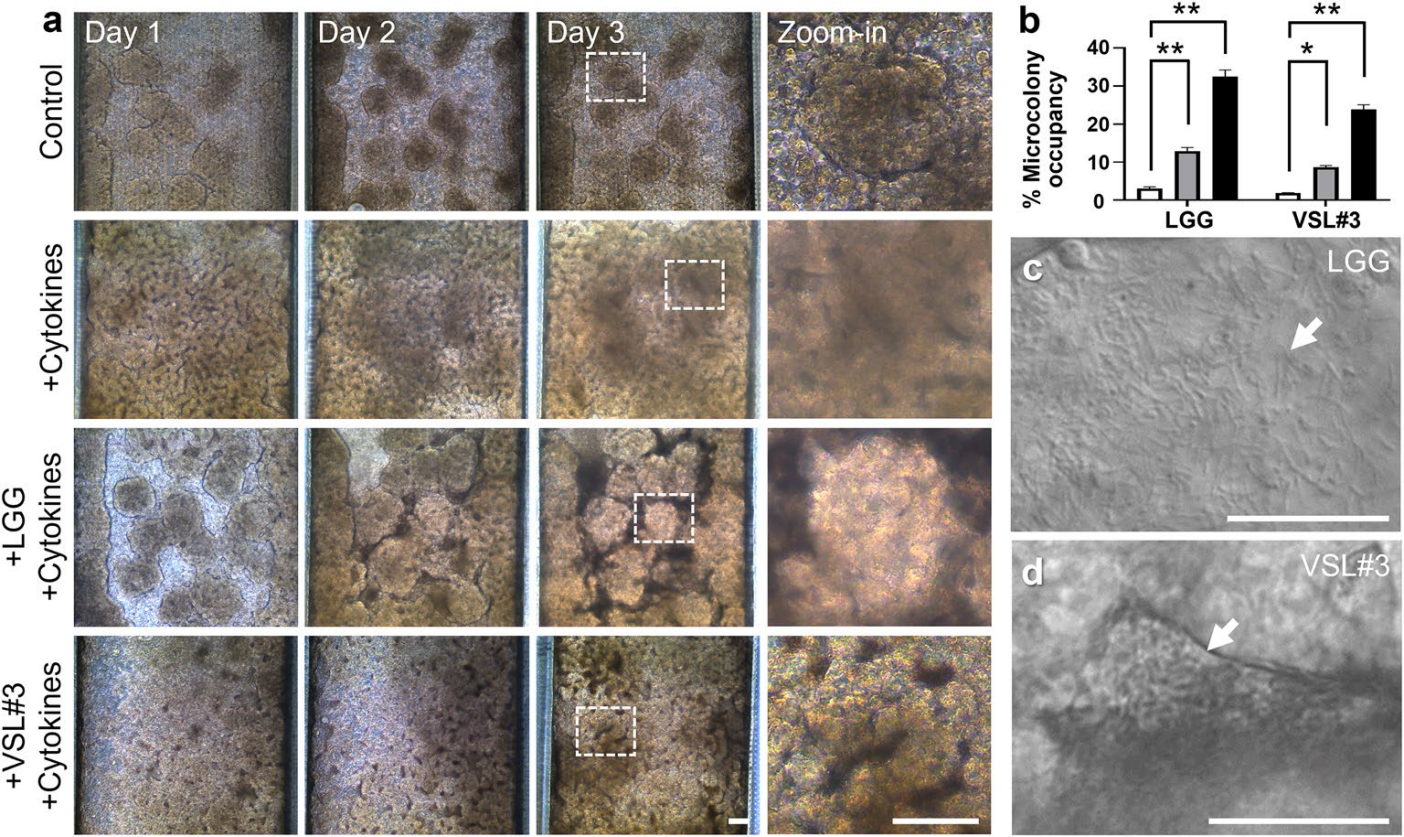
Morphological monitoring of the epithelial cells co-cultured with probiotic bacteria in a Leaky Gut Chip. (**a**) Phase-contrast micrographs of the morphological changes of epithelium challenged to a cytokine cocktail followed by the co-cultures with live LGG (+ LGG + Cytokines) and VSL#3 cells (+ VSL#3 + Cytokines) on days 1, 2, and 3. The image at “zoom-in” was enlarged from the location indicated in a dashed box in Day 3. (**b**) Quantification of the colonized area by either LGG or VSL#3 cells (% Microcolony occupancy) on the cytokine-challenged epithelium for 3 days (n = 3 per chip). The white, grey, and black columns indicate day 1, 2, and 3, respectively. A snapshot with a high-power magnification of LGG (**c**) or VSL#3 cells (**d**) co-cultured on a leaky epithelium. The DIC micrographs in (**c**) and (**d**) were captured from the real-time videos taken on Day 3 of the co-cultures provided in Supplementary Video S1 (LGG) and S2 (VSL#3), respectively. An arrow indicates the location of a microcolony. Bars, 50 µm. *p < 0.05, **p < 0.01.

In a pre-established germ-free Leaky Gut Chip, we colonized probiotic bacteria, either LGG or VSL#3, to interrogate if the probiotic intervention may contribute to restoring the compromised epithelial barrier. Once the intestinal epithelium was challenged to a cytokine cocktail for 3 days, the leaky epithelial layer was subsequently conditioned under an antibiotic-free, cytokine-free, and oxygen-controlled microenvironment for 24 h prior to a probiotic co-culture (Fig. 1a). Based on our established protocol for in vitro host–microbe co-cultures^24,26,28,32,33^ as well as the estimated maximum specific growth rate and doubling time (Supplementary Fig. S2 and Table S1), we performed longitudinal co-cultures of either LGG (Fig. 3a, + LGG + Cytokines) or VSL#3 (Fig. 3a, + VSL#3 + Cytokines) with the leaky intestinal epithelium for additional 3 days under a cytokine-free condition. For a stable and robust co-culture, we maintained the volumetric flow rate in a Leaky Gut Chip at 50 µL/h (corresponding shear stress, ~ 0.003 dyne/cm^2^), by which the dilution rate (*D*, 12.5 h^−1^) in a Leaky Gut Chip was at least 23 times higher than the maximum specific growth rate (µ_max_) of probiotic bacteria that we used in our study (Supplementary Table S1). As previously confirmed, the epithelium pre-exposed to a cocktail of pro-inflammatory cytokines reproducibly showed severe damage on its apical brush border and failed to restore intact epithelial morphology regardless of spontaneous remission after the cessation of cytokine treatment for additional 3 days (Fig. 3a, + Cytokines, Days 1–3 and Zoom-in). In contrast, the 3D epithelial layer that contained stochastic microcolonies of inoculated probiotics turned into an intact, restored mucosal morphology. When the leaky epithelium was colonized with a probiotic LGG strain or VSL#3 mixed strains, a clear outline of the villi-like 3D epithelium was observed in both low- and high-magnifications (Fig. 3a, + LGG + Cytokines or + VSL#3 + Cytokines). It is noted that the space between villi-like 3D epithelial layers was occupied by the colonized probiotic microcolonies, where the size of microcolonies progressively increased (Fig. 3b). Real-time monitoring of a colonized Leaky Gut Chip visualized highly viable probiotic bacterial cells of LGG (Fig. 3c and Supplementary Video S1) or VSL#3 (Fig. 3d and Supplementary Video S2) verified a stable host–probiotic ecosystem established in a Leaky Gut Chip without uncontrolled bacterial overgrowth.

### Probiotic co-culture induces epithelial barrier restoration in a Leaky Gut Chip

**Figure 4:**
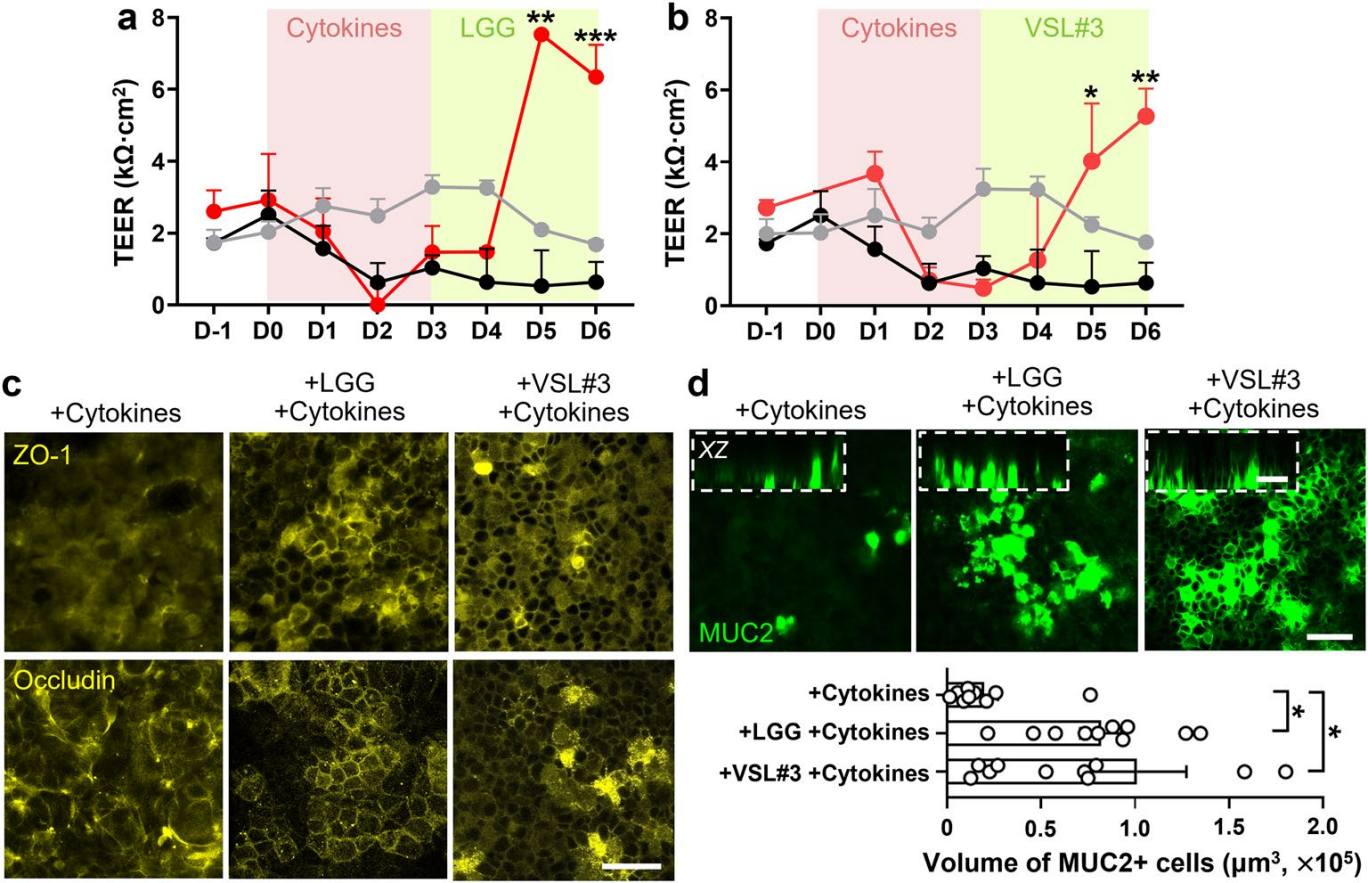
Restoration of impaired epithelial barrier when co-cultured with probiotic bacteria in a Leaky Gut Chip. The profile of epithelial barrier function measured by TEER when LGG (**a**) or VSL#3 bacterial cells (**b**) were co-cultured (red) in the cytokine-challenged Leaky Gut Chip compared to the cultures with cytokine challenges without probiotic co-cultures (black) or no cytokine challenges (grey). The pink and light green boxes indicate the period of the cytokine treatment and the probiotic co-culture, respectively. Cytokine treatment ceased during the probiotic co-cultures. The TEER profile of the Control and the Cytokine treated groups were replicated from Fig. 2d. *p < 0.05, **p < 0.01, ***p < 0.001. (**c**) Immunofluorescence visualization of the localization of ZO-1 and occludin in Leaky Gut Chips stimulated with probiotic bacteria (+ LGG + Cytokines or + VSL#3 + Cytokines) or germ-free controls without probiotic stimulation (+ Cytokines). Bar, 50 μm. (**d**) Immunofluorescence confocal micrographs of epithelial cells that highlight the expression of *MUC2* before (+ Cytokines) and after a probiotic co-culture with either LGG (+ LGG + Cytokines) or VSL#3 (+ VSL#3 + Cytokines) in a Leaky Gut Chip. An inset in a dashed box shows a cross-sectional vertical view (*XZ*) in the same chip analyzed. A bar chart displays the quantification of MUC2-positive cells in volumes as a function of probiotic co-cultures. *p < 0.05. Bars, 50 μm.

Next, we investigated probiotic contribution to the improvement of epithelial barrier function. After a 3-day-long treatment of a cytokine cocktail, probiotic bacteria (LGG or VSL#3) resuspended in an antibiotic- and oxygen-free medium were inoculated into the lumen compartment, then the probiotic effect on the barrier integrity was quantitatively examined by measuring the TEER of leaky epithelial layers (Fig. 1b). We found that both LGG and VSL#3 significantly increased TEER value up to 7.53 ± 0.09 (Fig. 4a) and 5.27 ± 1.08 kΩ cm^2^ (Fig. 4b), respectively, which displayed even higher than the one in the control group (maximum TEER at 3.28 ± 0.57 kΩ cm^2^). We further interrogated the expression and localization of tight junction proteins, ZO-1 and occludin. We found that the co-culture with either LGG or VSL#3 dramatically facilitated the rearrangement of ZO-1 at the cell–cell junction area in a Leaky Gut Chip, whereas the cytokine-challenged control showed scattered ZO-1 expression with minimal junctional localization (Fig. 4c, ZO-1 and Supplementary Fig. S3). Similarly, the co-culture with either LGG or VSL#3 respectively promoted the rearrangement of occludin at the cell junction area in a Leaky Gut Chip (Fig. 4c, Occludin and Supplementary Fig. S4). Moreover, the expression of mucin 2 (MUC2) was significantly upregulated (*p* < 0.05; Fig. 4d) when either LGG strain or VSL#3 was co-cultured with leaky epithelium for 3 days, whereas a lack of those probiotic bacteria resulted in minimal expression of MUC2 (Fig. 4d).

### Probiotic co-culture suppresses intestinal inflammation in a Leaky Gut Chip

**Figure 5:**
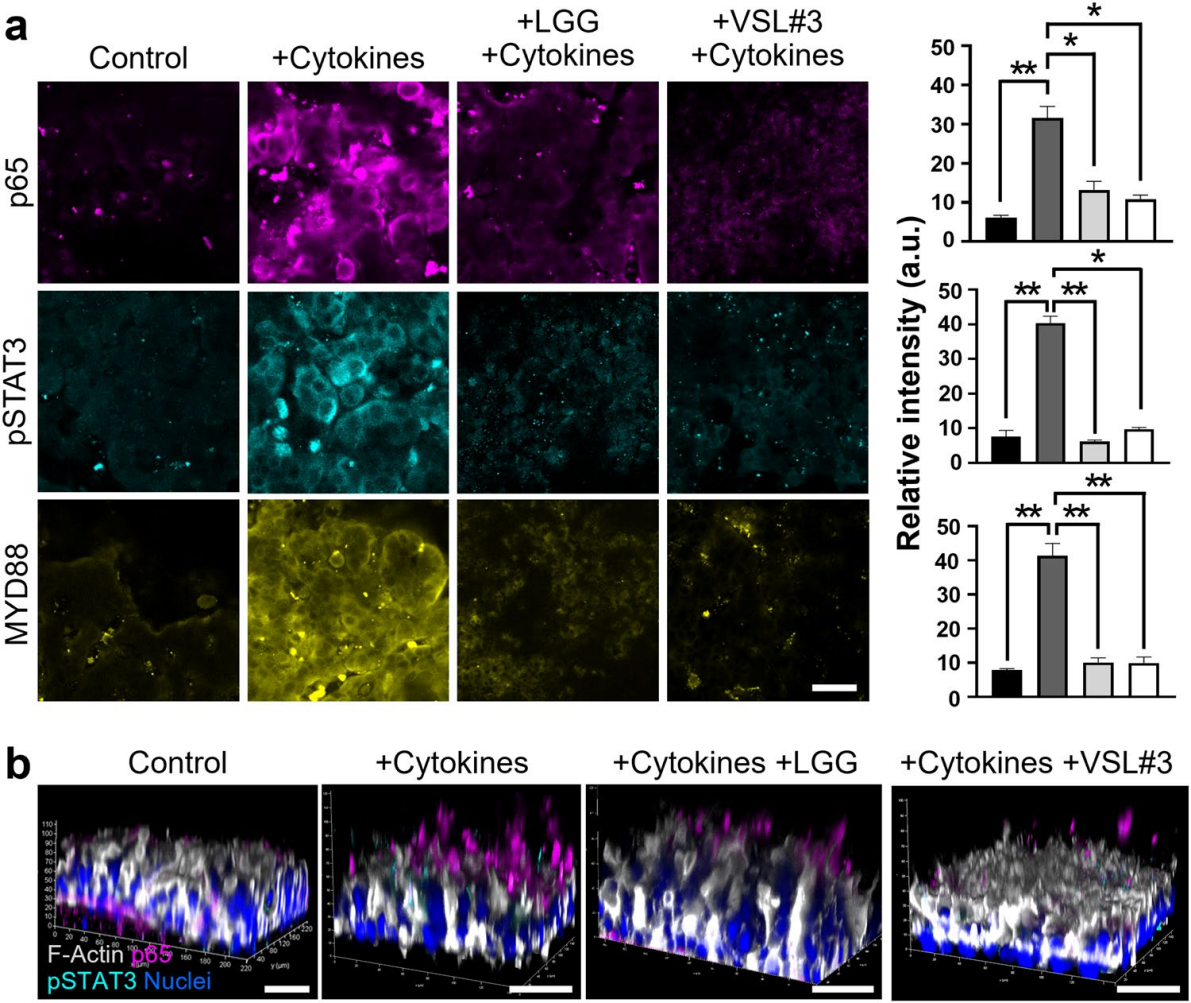
The anti-inflammatory effect of probiotic administration in a Leaky Gut Chip. (**a**) Probiotic co-cultures with either LGG (+ LGG, + Cytokines) or VSL#3 (+ VSL#3, + Cytokines) significantly reduced the expression of pro-inflammatory markers such as p65 (magenta), pSTAT3 (cyan), and MYD88 (yellow) compared to the cytokine-challenged group (+ Cytokines) on Day 3. A germ-free control that was not treated with a cytokine cocktail was used to provide a baseline expression of these three markers (Control). The column charts (right) show the quantification of the paired image. The black, dark grey, light grey, and white columns indicate Control, + Cytokines, + LGG + Cytokines, and + VSL#3 + Cytokines, respectively. Bar, 50 µm. *p < 0.05, **p < 0.01. (**b**) The 3D reconstruction of z-stacked images that visualizes the spatial distribution of p65 and pSTAT3 on the inflamed leaky epithelium elicited by cytokine challenges in a Leaky Gut Chip. Nuclei and F-actin were counter-stained with DAPI and fluorescence-labeled phalloidin, respectively. Bars, 50 µm.

We also evaluated the anti-inflammatory function of probiotic LGG or VSL#3 on the cytokine-challenged intestinal epithelium cultured in a Leaky Gut Chip. We performed an immunofluorescence confocal microscopic investigation that targets three representative inflammatory markers, including transcription factor p65 that encodes the *RELA* gene, one of the subunits of nuclear factor kappa-light-chain-enhancer of activated B cells (NF-κB)^34^, phosphorylated signal transducers and activator of transcription 3 (pSTAT3) transcription factor^35^, and myeloid differentiation primary response 88 (MYD88)^36^. When the intestinal epithelium in a Leaky Gut Chip underwent a cytokine pressure for 3 days followed by the cytokine-ceased remission for additional 3 days, the expression of p65, pSTAT3, and MYD88 was significantly upregulated (*p* < 0.01) compared to the control (Fig. 5a). Interestingly, when the leaky epithelium was colonized by either LGG or VSL#3 for 3 days after a cytokine challenge, the relative immunofluorescence intensity was significantly reduced (*p* < 0.05), suggesting that a probiotic co-culture positively regulates cellular inflammatory responses. This anti-inflammatory signature in response to probiotic co-cultures was also verified in 3D-rendered micrographs, where the epithelial cells with strong signals of p65 and pSTAT3 remarkably reduced when probiotic bacteria were co-cultured (Fig. 5b).

**Figure 6:**
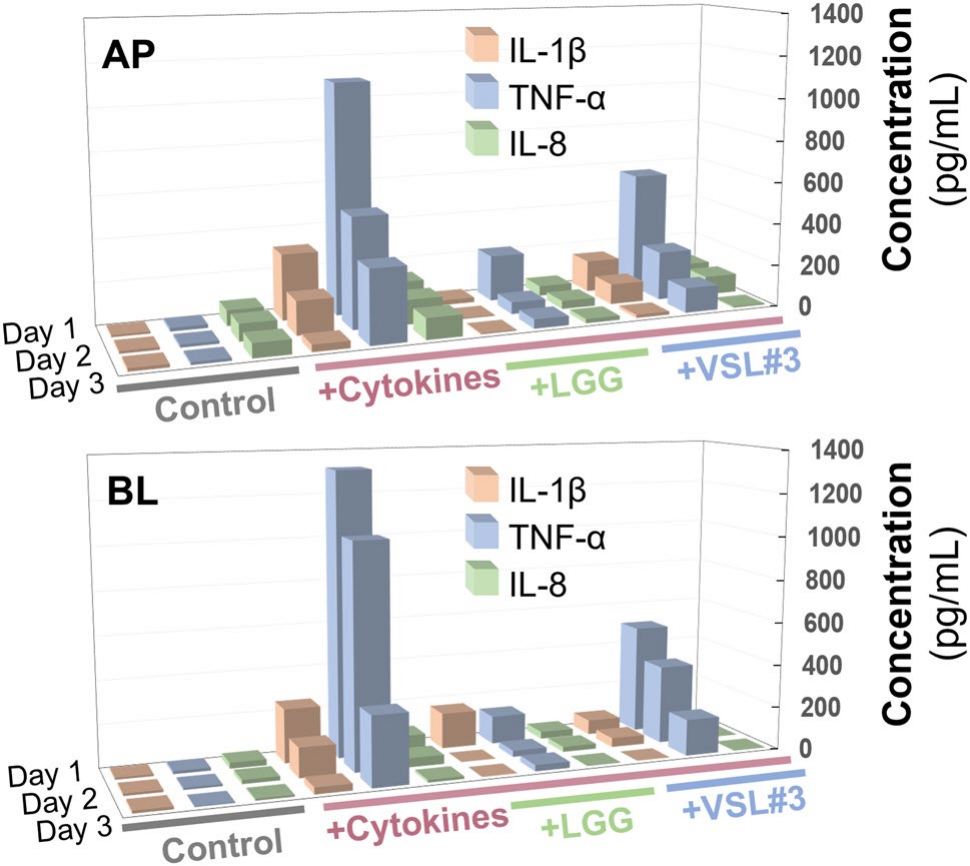
Extracellular secretion of pro-inflammatory cytokines, IL-1β, TNF-α, and IL-8, at various probiotic co-cultures on Days 1, 2, and 3. The colored lines indicate the group of samples that we assessed (Grey, Control; Red, + Cytokines; Green, + LGG + Cytokines; Blue, + VSL#3 + Cytokines). Samples were periodically collected from the apical (AP) or the basolateral compartment (BL), then quantitated. The concentration of secretory cytokines in the presence of probiotics (+ LGG + Cytikines or + VSL#3 + Cytokines) was significantly lower than those in the cytokine-challenged group (+ Cytokines) across the treated days in both compartments (*p* < 0.05 or lower). The Control group was challenged neither by cytokines nor probiotics. The raw data were provided in Supplementary Table S2.

We also quantitatively assessed the secreted pro-inflammatory cytokines such as IL-1β, TNF-α, and IL-8 produced by the epithelial cells when they were co-cultured with probiotic bacteria. After the cessation of cytokine treatment, we periodically collected the effluent from both apical and basolateral microchannels, then measured the amount of secretory cytokines by enzyme-linked immunosorbent assay (ELISA). When the leaky epithelial cells were in the remission stage without probiotic co-cultures, the level of secreted cytokines in both apical (AP) and basolateral (BL) channels was the highest on Day 1 of cytokine cessation (e.g., 134.07 ± 1.83, 1083.15 ± 28.11, and 322.42 ± 9.71 pg/mL for IL-1β, TNF-α, and IL-8 from apical effluents, respectively), which progressively decreased over 3 days (Supplementary Table S2). In contrast, when the leaky epithelium was colonized with either LGG or VSL#3, secretory pro-inflammatory cytokines of IL-1β, TNF-α, and IL-8 were significantly and progressively reduced to 19.92 ± 0.17, 44.57 ± 1.56, and 4.88 ± 0.76 pg/mL (Fig. 6, + LGG + Cytokines) or 6.92 ± 1.11, 117.96 ± 2.99, and 14.95 ± 0.35 pg/mL (Fig. 6, + VSL#3 + Cytokines) in Day 3 compared to the “+ Cytokines” group (Supplementary Table S2). The reduction of cytokine production was statistically significant when co-cultured regardless of the probiotic strains compared to the germ-free control with a cytokine challenge (Supplementary Table S2). However, the level of secretory inflammatory cytokines after probiotic co-cultures did not reach the baseline levels of cytokines in the Control group. Hence, we confirmed that the probiotic intervention with both LGG and VSL#3 probiotics effectively suppresses cytokine-induced epithelial inflammation.

## Discussion

This study reports a pathomimetic Leaky Gut Chip model that recapitulates leaky intestinal epithelial layers to validate barrier restoration and anti-inflammatory effects of live biotherapeutic probiotics. We leveraged a modular gut-on-a-chip microsystem to accurately manipulate luminal flow, peristalsis-like physical deformation, and oxygen gradient, in which stable colonization and maintenance of live probiotic bacteria were successfully demonstrated. Multi-modal imaging techniques such as real-time imaging of living bacterial cells and immunofluorescence confocal microscopy allow us to quantitatively assess pro- and anti-inflammatory responses of leaky epithelial cells under cytokine challenges and live biotherapeutic treatment, respectively. In addition, conventional bioelectrical and immunological assays confirmed the beneficial contribution of probiotic intervention in a Leaky Gut Chip.

The leaky gut syndrome is characterized by an abnormal increase of intestinal epithelial permeability. To investigate the effect of probiotic strains as live biotherapeutics, we minimized biological complexity to induce leaky gut symptoms by adding a cocktail of pro-inflammatory cytokines, IL-1β and TNF-α. It is noted that these two cytokines are potent to induce impaired epithelial barrier and an inflammatory cascade that simulates a pathophysiological cytokine milieu in the mucosal interface^37–40^. Our experimental modeling targeted to capture an essential determinant of epithelial barrier dysfunction and inflammatory microenvironment. Multiple studies have reported that IL-1β promotes intestinal inflammation via the disruption of intestinal epithelial barriers followed by infiltration of luminal antigens into the lamina propria^39,41^. Similarly, TNF-α has been known to independently induce barrier disruption^30,37^, decrease ZO-1 expression^42,43^, and elicit epithelial inflammation^44^. Some studies may require an IFN-γ pretreatment^45^, which is not always necessary when the concentration of TNF-α is high enough (i.e., > 10 ng/mL)^30,37^. Importantly, a synergistic pro-inflammatory effect under a co-stimulation with TNF-α and IL-1β was supported by multiple prior reports that illustrated an elevated expression of p65^43,46^, pSTAT3^47^, and MYD88^48,49^, which was replicated in our study.

To induce leaky epithelium, we treated a cytokine cocktail for 3 days, then ceased the treatment. There are several reasons for the cessation of a cytokine cocktail in the current study. First, the optimized condition of cytokine treatment applied in this study showed a significant but non-catastrophic loss of epithelial barrier, 3D morphology, tight junctions and permeability, and mucus production. Thus, it was not necessary to continue challenging the epithelium with a high level of pro-inflammatory cytokines in a given condition. Second, while IL-1β and TNF-α are potent pro-inflammatory cytokines that induce leaky barrier and intestinal inflammation, they are also the representative secretory cytokines that the inflamed epithelium releases. Thus, we ceased the treatment of IL-1β and TNF-α, by which representative cytokines produced by the inflamed epithelium are characterized without contamination by the pre-administered cytokines. Finally, when an administration of probiotics may induce anti-inflammatory responses and restoration of epithelial barrier integrity, it was expected that the production of pro-inflammatory cytokines would also be significantly diminished^50–52^. To better simulate this pathophysiological condition, we ceased the cytokine treatment.

Our Leaky Gut Chip model reproducibly replicated a compromised 3D morphology with disrupted cell–cell junction as well as a loss of epithelial permeability barrier and dysregulated junctional proteins under physiological conditioning by treating both TNF-α and IL-1β. We confirmed in our Leak Gut Chip that the TEER measurement was indicative of the temporal profile of barrier dysfunction and its restoration as a function of probiotic co-cultures. Several factors can elevate the TEER value during probiotic co-cultures, evidenced by the rearrangement of tight junction and adherence junction proteins, accumulation of mucus and gel-forming MUC2 proteins, and increased occupancy of colonized probiotic bacterial cells on the mucosal surface. Our previous studies confirmed that the administration of probiotic bacteria alone, such as *Lactobacillus rhamnosus* GG^24^ and VSL#3^32^, without any pro-inflammatory mucosal stimulation, gradually but significantly enhanced the barrier function of Caco-2 intestinal epithelium quantified by TEER while observing no evidence of leaky epithelial barrier, impaired epithelial morphology, and inflammatory epithelial responses. Based on this preliminary study, we predominantly focused on the validation of the effect of probiotic bacteria on the amelioration of intestinal inflammation and the restoration of epithelial barrier integrity.

Our findings suggest that both LGG and VSL#3 significantly reduced the expression of inflammatory signaling molecules (p65, pSTAT3, and MYD88) in the intestinal epithelium cultured in a Leaky Gut Chip. This observation shows a good agreement with previous reports. For instance, probiotics can decrease IL-6 and IL-1β production by modulating the NF-κB and STAT3 pathways under inflamed conditions^53^. In particular, NF-κB is composed of several protein subunits, which regulate the transcription of effector genes associated with the production of pro-inflammatory cytokines TNF-α and IL-1β^39,43,54^. The activation of STAT3 reflects the inflammatory epithelium challenged by pro-inflammatory cytokines^55^, where pSTAT3 in intestinal epithelial cells was predominantly detected upon epithelial injury in a dextran sodium sulfate (DSS)-mediated colitis mouse model^56^. MYD88 is an essential toll-like receptor (TLR) mediator that activates the NF-κB pathway and produces critical cytokines (e.g., IL-1β, IL-12, and IL-17A)^57^ and antimicrobial peptides (e.g., RegIIIγ)^58^ involved in the maintenance of the intestinal barrier in the host–microbiome interface. Thus, the elevated expression of these transcription factors (p65 and pSTAT3) and a cellular mediator (MYD88) followed by the extracellular secretion of pro-inflammatory cytokines are hallmarks of epithelial inflammatory responses in a Leaky Gut Chip. The probiotic co-culture remarkably reduced the expression of p65, pSTAT3, and MYD88 along with a significant decrease of secretory IL-1β, TNF-α, and IL-8, suggesting that the microbiome-mediated anti-inflammatory regulation was successfully demonstrated in a Leaky Gut Chip. Our Leaky Gut Chip model confirms the anti-inflammatory effects of probiotics consistent with outcomes of former studies^59–62^.

It is noted that the impaired epithelial barrier may cause problematic bacterial translocation from the lumen to the capillary side, which may exacerbate intestinal inflammation^28^. From a clinical standpoint, translocation of luminally administered probiotics may cause serious adverse effects such as sepsis and septic shock^28,63,64^. In our study, we did not detect any bacterial translocation from the lumen to the abluminal compartment channel in a Leaky Gut Chip evidenced by no bacterial growth in the collecting tube linked to the basolateral microchannel over co-cultures regardless of the cytokine treatment. We may suggest an intriguing study in the future, where the severity of barrier dysfunction can be adjusted, then different types of probiotic bacteria can be tested if they may behave in a beneficial or an opportunistic manner depending on the degree of barrier impairments. This idea can be further advanced by applying patient-derived organoid epithelium and the paired immune cells from the same donor, which may allow us to better determine the dose, types of probiotics, the necessity of prebiotics, and the effect of post-biotics (e.g., short-chain fatty acids).

Living bacterial cells often overgrow in static in vitro cultures^65^. To avoid an overgrowth of live probiotic bacteria in a Leaky Gut Chip, we harnessed the microfluidic flow cell system as a continuous plug flow reactor (PFR) in a device by manipulating the steady-state condition inside the luminal and abluminal microchannel. The dilution rate calculated in our Leaky Gut Chip is approximately 12.5 h^−1^, which was obtained by dividing the applied volumetric flow rate by the estimated volume of the upper microchannel. Notably, the applied dilution rate is much higher than the maximum specific growth rate of LGG (see Supplementary Table S1; 0.296 h^−1^), indicating that bacterial overgrowth can be effectively controlled in our system. It is also noted that the colonized probiotic bacteria on the mucosal surface, especially at the intervillous crevice, supplement the repopulation of probiotic bacterial cells in a Leaky Gut Chip. This condition can dampen the bacterial overgrowth in the chip and lead to continuous host–probiotic interactions while prolonging the co-culture period to 3 days or even longer. Therefore, it was not necessary to specifically determine a dose-dependent seeding effect because bacterial cells progressively increase their number and reach a saturation point at a given volumetric flow rate (also at a defined dilution rate) under this pseudo-turbidostat condition. As noted, the bacterial seeding density was experimentally determined to be ~ 10^7^ colony forming units (CFU)/mL as previously established^24,32^.

We recognize possible limitations for the translational implementation of our current study. First, our model specifically simulates the mucosal microenvironment colonized by the probiotics on the apical brush border of 3D epithelium in a Leaky Gut Chip. Thus, a macroscopic milieu of probiotic administration (e.g., luminal vs. mucosal probiotic disposition) may not be fully demonstrated. Second, our Leaky Gut Chip exclusively demonstrates the microenvironment at a certain location of the small or large intestine but does not model the feature beyond these organs. Thus, the possible loss of orally administered probiotics by gastric juice with extremely low pH, chemical attack by bile acids and their conjugates, or enzymatic damage by digestive enzymes was not accurately emulated in our current system. Third, we did not add patient-derived fecal microbiota or any relevant luminal or mucosal multi-species microbiota in the current system. Thus, the effect of administered probiotics in a leaky epithelial barrier in a clinical setting may require additional follow-up studies by incorporating patient-derived epithelium, paired fecal or mucosal microbiota, and potentially immune cells as well.

The Leaky Gut Chip used in this study can independently couple individual interacting factors (i.e., epithelium, inflammatory cytokines, and probiotic bacteria) to verify the contribution of probiotic treatment to the restoration of the impaired epithelial barrier and inflamed mucosa. Furthermore, our strategy may be disseminated to appeal for a personalized Leaky Gut Chip by integrating patient-derived intestinal organoid epithelium and fecal microbiota, as previously mentioned. Indeed, this approach will offer a novel avenue to reflect individual donors’ genetic and pathophysiological backgrounds. The modularity of the Leaky Gut Chip ultimately allows us to challenge the leaky epithelial layer in germ-free (i.e., the cells of the host alone), gnotobiotic (i.e., the administration of a probiotic strain) and colonized microbial milieus (i.e., a co-culture of both probiotics and fecal microbiome) in a combinatorial manner. Hence, if different host cells and probiotics are matched appropriately, we can produce a disseminating database of epithelial regeneration, which is valuable for precision medicine understanding. Finally, a Leaky Gut Chip can potentially be implemented for screening “Druggable probiotics” to repurpose first-generation probiotics (FGP) or next-generation probiotics (NGP) for precision probiotic therapeutics^66^. Therefore, using the Leaky Gut Chip model, it is possible to select the most appropriate strain of probiotics to be taken for a longer period of time with high compliance, or possibly a genetically-engineered strain to be used to treat gastrointestinal disorders.

## Methods

### Microfabrication of a Gut Chip

Silicon molds for making a 500-μm-high upper microchannel and a 200-μm-high lower microchannel were created using standard photolithography techniques as previously described^24,29^. Next, a mixture of polydimethylsiloxane (PDMS) at a 15:1 ratio (w/w) of silicone elastomer:curing agent (Sylgard 184, Dow Corning) was degassed and poured onto a dried mold, then cured in a dry oven at 60 °C for > 4 h. The cured PDMS was then demolded and punched (2 mm in diameter; Harris Uni-Core, GE Healthcare) to create connecting ports to microchannels. A PDMS porous membrane was obtained using a metal wafer patterned with an array of etched micro-pillars (10 µm in diameter of the circle of a pillar, 25 µm spacing from the center of a circle to another circle, 25 µm in height; Applied Novel Devices). Briefly, a degassed PDMS (~ 10 g) was poured on a dried membrane wafer, on which a polyester film (3 M Medical Materials and Technologies) was placed without air bubble, a flat PDMS slab and a 3-kg weight were sequentially stacked up to provide gravitational compression, then a whole setup was incubated at 60 °C overnight. To bond each microchannel layer and a porous membrane, the surface of an upper layer and a porous membrane patterned on a polyester film was activated with a plasma cleaner (COVANCE; Femto Science Inc.) for 75 s (125 W, 20 Pa), bonded by facing the activated PDMS surface in a conformal angle, and incubated in a dry oven (Thermo Fisher Scientific) at 70 °C overnight. After peeling the upper layer-membrane set off from the polyester film, this part was accurately aligned to the lower PDMS microchannel layer under a stereoscope (MDG41; Leica Microsystems) after surface activation using a plasma cleaner with the same setting. To create inlets and outlets in a device, an 18-gauge blunt-end needle (Shintop) was bent at a 90° angle, cut, linked into a 2-inch-long silicone tubing (Tygon 3350, ID 1/32", OD 3/32", Saint-Gobain), and inserted into the punched hole to make a complete microfluidic device.

### Microfluidic cell culture

Human intestinal epithelial Caco-2BBE cells (Harvard Digestive Disease Center) were cultured in a T75 tissue culture flask in Dulbecco's Modified Eagle Medium (DMEM, Gibco) supplemented with 20% (v/v) fetal bovine serum (FBS; Thermo Fisher Scientific) and 100 U/mL penicillin (Gibco) and 100 μg/mL streptomycin (Gibco). When the Caco-2 cells reached ~ 90% confluency, cells were harvested by treating 0.25% trypsin and 0.02% (w/v) ethylenediaminetetraacetic acid (EDTA) solution (Thermo Fisher Scientific), resuspended with the culture medium at a single-cell level (final density, 5 × 10^6^ cells/mL), then introduced into the upper microchannel of a Gut Chip pre-coated with 1% (v/v) Matrigel (Thermo Fisher Scientific) and 30 μg/mL collagen type I (Thermo Fisher Scientific) extracellular matrix (ECM) on a pre-activated surface by UV/ozone treatment (Jelight Company Inc.) for 1 h. After cell attachment in a humidified, 5% CO_2_ incubator at 37 °C for 1 h, the device set was connected to a syringe pump (Braintree Scientific) and perfused at a constant flow rate of 30 μL/h to the upper microchannel for the first day, then switched at 50 μL/h (corresponding fluid shear stress, ~ 0.003 dyne/cm^2^) from the second day onward to both upper and lower microchannels. Once the epithelial cells formed an intact tight junction barrier on-chip, mechanical stretching motions (10% in cell strain, 0.15 Hz in frequency) were applied by connecting the vacuum chambers to a vacuum line under a computer-controlled cyclic tensile load (Flexcell International Corporation). We maintained this physiological culture condition of flow and deformation until the end of cytokine treatment as well as co-cultures.

### Induction of leaky gut condition

Once Caco-2 cells form a 3D epithelial microarchitecture in a Gut Chip, we reduced the FBS level in the culture medium from 20 to 5% (v/v) to control cell proliferation. Next, we perfused the filter-sterilized (cutoff size, 0.22 μm) culture medium that contains 5% FBS, antibiotics, and a cocktail of TNF-α (100 ng/mL; PeproTech) and IL-1β (100 ng/mL; PeproTech) for 3 days. After 48 h since the cytokine treatment, we switched the aforementioned medium to the antibiotic-free medium to both upper and the lower microchannels at least 24 h prior to the inoculation of probiotic bacteria. We added l-cysteine (final concentration at 1 mg/mL; Sigma-Aldrich) in this medium and flowed into only the upper microchannel to create an oxygen gradient in a Leaky Gut Chip until the end of the co-cultures. After cytokine treatment for 72 h, the cytokine cocktail was removed from the culture medium, then probiotic co-culture was performed.

### Probiotic co-culture in a Leaky Gut Chip

To perform longitudinal host–probiotic co-cultures in a Leaky Gut Chip, we used two live biotherapeutic products, *Lactobacillus rhamnosus* GG (ATCC 53103) and over-the-counter VSL#3 formulation (Sigma-Tau Pharmaceuticals, Inc.). For an LGG pre-culture, we grew LGG cells in a 15-mL sterilized test tube containing 3 mL autoclaved Lactobacilli MRS Broth (MRS; Difco). For a VSL#3 pre-culture, we inoculated VSL#3 power in the mixture (3 mL) of autoclaved MRS broth and Reinforced Clostridial Medium (RCM; Difco) at 1:1 (v/v) in a 15-mL sterilized tube. Next, we incubated the inoculated test tubes in a GasPak EZ container (BD Diagnostics) containing two anaerobic gas-generating GasPak EZ sachets (BD Diagnostics) without shaking for 12–18 h. After a seed culture, we centrifuged the culture broth at 10,000×*g* for 1 min, aspirated the supernatant, resuspended the cell pellet with an antibiotic-free, l-cysteine-containing culture medium and adjusted the final cell density at 1 × 10^7^ CFU/mL. Using a 200P micropipette, we inoculated bacterial cells into the upper microchannel of a germ-free Leaky Gut Chip that was pre-conditioned with an antibiotic-free, AOI-created culture microenvironment, then incubated the setup without flow at 37 °C for 1 h. After incubation, we resumed the flow (50 μL/h) and physical deformations until the experiments were finished.

### Assessment of epithelial barrier function

The epithelial barrier integrity was assessed by measuring transepithelial electrical resistance (TEER). We inserted two Ag/AgCl electrodes (A–M Systems) into the upper and the lower microchannels, respectively, to measure a resistance value of an epithelial layer using a multimeter (Fluke Corporation). The TEER value (kΩ cm^2^) was calculated as follows; TEER = (Ω_t_ − Ω_blank_) × A, where Ω_blank_ is the resistance in the blank chip without epithelium (measured prior to Caco-2 seeding), Ω_t_ is the resistance at the time point of interest, and A is the area of the surface covered by a cell layer. The cell culture area in a Gut Chip used in this study was approximately 0.11 cm^2^. Apparent permeability was evaluated by flowing a culture medium that contained 100 μg/mL of fluorescein sodium salt (376.27 Da; Sigma) to the upper channel at 50 μL/h while the lower microchannel flowed with a blank culture medium at the same flow rate. After the effluent from the lower microchannel was collected every 30 min, fluorescence intensity (excitation at 494 nm, emission at 512 nm) was measured using a microplate reader (Molecular Devices). The concentration of the transported fluorescein was determined by a standard curve. The apparent permeability (cm/s) was calculated using the following equation; *P*_app_ = dQ/dt × 1/(A × C_0_), where dQ is the amount of transported fluorescein (μg), dt is the time interval (s), A is the area of the membrane surface (cm^2^), and C_0_ is the original concentration of fluorescein in the upper channel (μg/cm^3^)^67^.

### Microscopic analysis

The morphology of intestinal epithelium was monitored with phase-contrast or differential interference contrast (DIC) microscopy by using either an inverted phase-contrast microscope (DMi1, Leica Microsystems) equipped with a 10× or a 20× objective (NA 0.22 or NA 0.3, respectively; Leica Microsystems) and a camera (1824 × 1368, MC120 HD; Leica Microsystems) or a confocal microscope (DMi8, Leica Microsystems) equipped with a 25× objective (NA 0.95; water immersion; Leica Microsystems) and a camera (1920 × 1440; sensor, Sony ICX674AQG CCD; Leica Microsystems). Images were acquired on either the LAS EZ (DMi1) or the LAS X (DMi8) software (Leica Microsystems). The stitched images were obtained with a 10× objective (NA 0.30, Leica Microsystems) taken automatically using the LAS X function Define Tilescan Experiment. Fluorescence images were acquired using the aforementioned confocal microscope equipped with a TCS SPE confocal system holding four solid-state excitation laser sources of 405 nm, 488 nm, 532 nm, and 635 nm and an Ultra-high dynamic PMT detector (Leica Microsystems). Real-time live recordings of living microbial cells were obtained in the DIC mode in a DMi8 microscope (25× water immersion objective; 1920 × 1440 live format; 20 frames per second).

To perform immunofluorescence staining, a Leaky Gut Chip that contains target host cells was fixed with 4% (w/v) paraformaldehyde (PFA; Electron Microscopy Sciences) for 30 min, permeabilized with Triton X-100 (0.3%, v/v; Sigma) for 30 min, then blocked with filter-sterilized 2% (w/v) bovine serum albumin (BSA; Hyclone Laboratories Inc.) for 1 h. All the steps were performed at room temperature. Each reagent (20 µL) was infused into both microchannels using a micropipette. Phosphate-buffered saline (PBS; Ca^2+^- and Mg^2+^-free) was used as a washing buffer in the microchannel between the steps. Next, an appropriate combination of primary antibodies was diluted in 2% BSA. Primary antibodies of anti-ZO-1 (61-7300; Thermo Fisher Scientific), anti-p65 (ab16502; Abcam), anti-MYD88 (PA519919; Thermo Fisher Scientific), anti-pSTAT3 (9138S; Cell Signaling Technology), or anti-Occludin (sc-133256; Santa Cruz Biotechnology) were used. The primary antibody solution was introduced into both microchannels under a static condition and incubated at room temperature for 1 h, then additionally incubated at 4 °C overnight. After the chips were washed with PBS, an appropriate combination of secondary antibodies was diluted in 2% BSA. Secondary antibodies of goat anti-mouse IgG DyLight 488 (ab96871; Abcam), goat anti-rabbit IgG DyLight 488 (ab96883; Abcam), donkey anti-mouse IgG DyLight 555 (ab150106; Abcam), goat anti-rabbit IgG DyLight 555 (ab150078; Abcam), or goat anti-mouse IgG DyLight 650 (ab96882; Abcam) were used. The secondary antibody solution was infused into both microchannels without flow for 1 h, then incubated at room temperature under light protection for another hour. To visualize F-actin and nuclei, Alexa Fluor 647 Phalloidin (330 nM, final concentration; Thermo Fisher Scientific) and 4′,6-diamidino-2-phenylindole dihydrochloride (DAPI; 5 µg/mL, final concentration; Thermo Fisher Scientific), respectively, were introduced for 30 min following the secondary antibody immunolabeling.

The 3D rendered images were prepared by processing Z-stacked images using Leica LAS X software. To obtain the size of microcolonies, we used the Freehand Selections tool in ImageJ to draw boundaries around the microcolonies on the phase-contrast images over three co-culture days. The enclosed areas were then calculated using a Measure tool and normalized by the area of total epithelial occupancy captured in a micrograph. To quantify the volume of the MUC2-positive region in a 3D rendered reconstruction file, we processed Z-stacked immunofluorescence images using IMARIS software (version 8.4.1; Bitplane). A surface object was created without smoothing for the 3D reconstructed image that highlights the MUC2 signal, then the 3D volume of MUC2-positive signals (unit, μm^3^) was determined after adjustment of the intensity threshold. The relative intensity of fluorescence micrographs was quantitatively evaluated using ImageJ.

### Measurement of secretory cytokines

We intermittently collected apical and basolateral effluents from both the upper and the lower microchannels at 50 µL/h for 4 h, respectively, centrifuged the collected sample to remove cell debris, and stored the supernatant immediately at − 80 °C until use. Cytokine quantification was performed using ELISA kits (Thermo Fisher Scientific) for TNF-α, IL-1β, and IL-8 based on the manufacturer protocol.

### Statistical analysis

All data and error bars in the article are represented as mean ± standard error of the mean (SEM). We applied a two-tailed unpaired *t*-test (Figs. 2, 3 and 4), a one-way analysis of variance (ANOVA) followed by a Tukey test (Fig. 5), or a two-way ANOVA followed by a Tukey test (Fig. 6). All statistical analyses were carried out using GraphPad Prism 9.3.1 (GraphPad Software). Differences between groups were considered statistically significant when *p* < 0.05.

## Supplementary Information

Supplementary Information 1.

Supplementary Video S1.

Supplementary Video S2.

## Data Availability

Data and source code are available upon a reasonable request to the corresponding author.
