# Supplementary material for: Live probiotic bacteria administered in a pathomimetic Leaky Gut Chip ameliorate impaired epithelial barrier and mucosal inflammation
Source: Sci Rep. 2022 Dec 31;12:22641. doi: 10.1038/s41598-022-27300-w (PMC9805460; doi:10.1038/s41598-022-27300-w)
Supplement: Supplementary file 1 — Supplementary Information 1. [file 41598_2022_27300_MOESM1_ESM.docx]

**SUPPLEMENTARY INFORMATION (SI)**

**Live probiotic bacteria administered in a pathomimetic Leaky Gut Chip ameliorate impaired epithelial barrier and mucosal inflammation**

Soyoun Min^1^, Nam Than^1,2^, Yong Cheol Shin^1^, Grace Hu^2^, Woojung Shin^3,4^, Yoko M. Ambrosini^5^, and Hyun Jung Kim^1,*^

^1^Department of Inflammation and Immunity, Lerner Research Institute, Cleveland Clinic, Cleveland, OH 44195, USA

^2^Department of Biomedical Engineering, The University of Texas at Austin, Austin, TX 78712, USA

^3^Wyss Institute for Biologically Inspired Engineering, Harvard University, Boston, MA 02115, USA

^4^Institute for Medical Engineering and Science, Massachusetts Institute of Technology, Cambridge, MA 02139, USA

^5^Department of Veterinary Clinical Sciences, College of Veterinary Medicine, Washington State University, Pullman, WA 99164, USA

*Soyoun Min and Nam Than contributed equally.*

***Correspondence to:**

Hyun Jung Kim, PhD

Department of Inflammation and Immunity

Lerner Research Institute

Cleveland Clinic

9500 Euclid Ave., NE3

Cleveland, OH 44195, USA

Phone: 216-445-8148

Email: [kimh19@ccf.org](mailto:kimh19@ccf.org)

**SI MATERIALS AND METHODS**

**Microbial growth profile.** To monitor the growth profile of LGG bacteria, we inoculated the pre-cultured LGG cells in an MRS medium that are resuspended in an antibiotic-free cell culture medium (with 5% FBS) or an MRS medium at an optical density of 0.05 at 600 nm (N=5) in a 96-well plate. We then incubated the well plate at 37^o^C in a microplate reader (Molecular Devices) set to measure the optical density at 600 nm (OD_600_) every 15 minutes for up to 18 h. The respective blank OD value at 600 nm of each medium was subtracted from the OD_600_ values in the test wells. The maximum specific growth rate (μ_max_, Supplementary Table S1) was determined by performing a log transformation of the OD_600_ values and calculating the slope of the most linear and steepest region in the transformed curves. The doubling time (T_D_) was calculated as T_D_ = ln(2)/μ_max_.

**Line scan analysis.** To perform a line scan analysis, we transformed the original fluorescence images into 16-bit grayscale, then used a linear drawing tool to indicate the target location and attain the intensity profile using ImageJ (Ver. 1.53f51).

**Real-time live recording**. The movie of live LGG or VSL#3 bacterial cells co-cultured in a Leaky Gut Chip was recorded using differential interference contrast (DIC) microscopy (DMi8, Leica Microsystems) equipped with a 63× objective (NA 1.4; oil immersion; Leica Microsystems) and a high-resolution camera (DFC7000 T; Leica Microsystems). Videos were acquired with the LAS X (DMi8) software (Leica Microsystems) at a rate of 12 frames/s under the Default Dynamic Widefield Tree configuration (laser and detector off).

**Legends of Supplementary Tables, Figures, and Videos**

**Supplementary Table S1.** The maximum specific growth rate and the doubling time of LGG bacterial cells cultured in either the MRS or the cell culture medium

| Medium | Maximum specific growth rate, *µ*_max_ (h^-1^) | Doubling time, τ_D_ (h) |
| --- | --- | --- |
| MRS | 0.53 | 1.32 |
| 5% DMEM | 0.30 | 2.34 |

*DMEM was supplemented with 5% (v/v) FBS.

**Supplementary Table S2.** The concentrations of TNF-α, IL-1β, and IL-8 collected from apical (AP) or basolateral (BL) microchannels at different experimental conditions

| Conc. (pg/mL) | | AP | | | BL | | |
| --- | --- | --- | --- | --- | --- | --- | --- |
|  |  | IL-1β | TNF-α | IL-8 | IL-1β | TNF-α | IL-8 |
| Day 1 | Control | 73.46  ± 1.83^c^ | 16.76  ± 0.58^c^ | 9.74  ± 0.69^c^ | 27.6  ± 0.81^c^ | 13.57  ± 0.24^c^ | 9.73  ± 0.58^c^ |
|  | +Cytokines | 134.07  ± 1.83 | 1083.15  ±28.11 | 322.42  ± 9.71 | 75.84  ± 0.49 | 1316.86  ± 9.78 | 254.13  ± 5.36 |
|  | +Cytokines  +LGG | 47.03  ± 0.79^c^ | 226.55  ± 3.76^c^ | 22.17  ± 1.28^c^ | 33.81  ± 0.91^c^ | 128.55  ± 1.16^c^ | 166.03  ± 4.3^c^ |
|  | +Cytokines  +VSL#3 | 93.39  ± 2.33^c^ | 566.72  ± 8.11^c^ | 148.49  ± 2.62^c^ | 23.98  ± 1.12^c^ | 518.47  ± 6.38^c^ | 68.7  ± 1.01^c^ |
| Day 2 | Control | 80.43  ± 2.14^c^ | 12.43  ± 0.68^c^ | 12.87  ± 0.88^c^ | 18.81  ± 0.49^c^ | 9.81  ± 0.3^c^ | 12.25  ± 1.07^c^ |
|  | +Cytokines | 116.69  ± 1.69 | 526  ± 19.1 | 159.56  ± 12.79 | 46.52  ± 2.49 | 1011.74  ± 0.96 | 137.59  ± 1.54 |
|  | +Cytokines  +LGG | 39.06  ± 0.78^c^ | 55.18  ± 3.28^c^ | 5.67  ± 1.1^c^ | 25.44  ± 2.13^c^ | 31.29  ± 1.35^c^ | 0.23  ± 0.06^c^ |
|  | +Cytokines  +VSL#3 | 86.57  ± 2.41^c^ | 229.2  ± 8.8^c^ | 94.08  ± 2.24^c^ | 1.28  ± 0.15^c^ | 373.95  ± 5.52^c^ | 44.27  ± 2.09^c^ |
| Day 3 | Control | 65.26  ± 1.28^c^ | 12.07  ± 1.05^c^ | 14.17  ± 0.94^b^ | 8.3  ± 0.28^ns^ | 4.28  ± 0.45^c^ | 12.35  ± 0.22^c^ |
|  | +Cytokines | 100.49  ± 4.33 | 334.59  ± 8.98 | 35.67  ± 3.24 | 11.63  ± 0.73 | 316.87  ± 1.94 | 30.27  ± 2.14 |
|  | +Cytokines  +LGG | 19.92  ± 0.17^c^ | 44.57  ± 1.56^c^ | 4.88  ± 0.76^c^ | 2.45  ± 0.37^c^ | 29.89  ± 0.57^c^ | 0.4  ± 0.19^c^ |
|  | +Cytokines  +VSL#3 | 6.92  ± 1.11^c^ | 117.96  ± 2.99^c^ | 14.95  ± 0.35^a^ | 1.4  ± 0.39^c^ | 165  ± 3.03^c^ | 0.2  ± 0.07^c^ |

*Cytokine levels were quantified by ELISA.

*a: *p*<0.5, b: *p*<0.01, c: *p*<0.001, ns: not significant

**
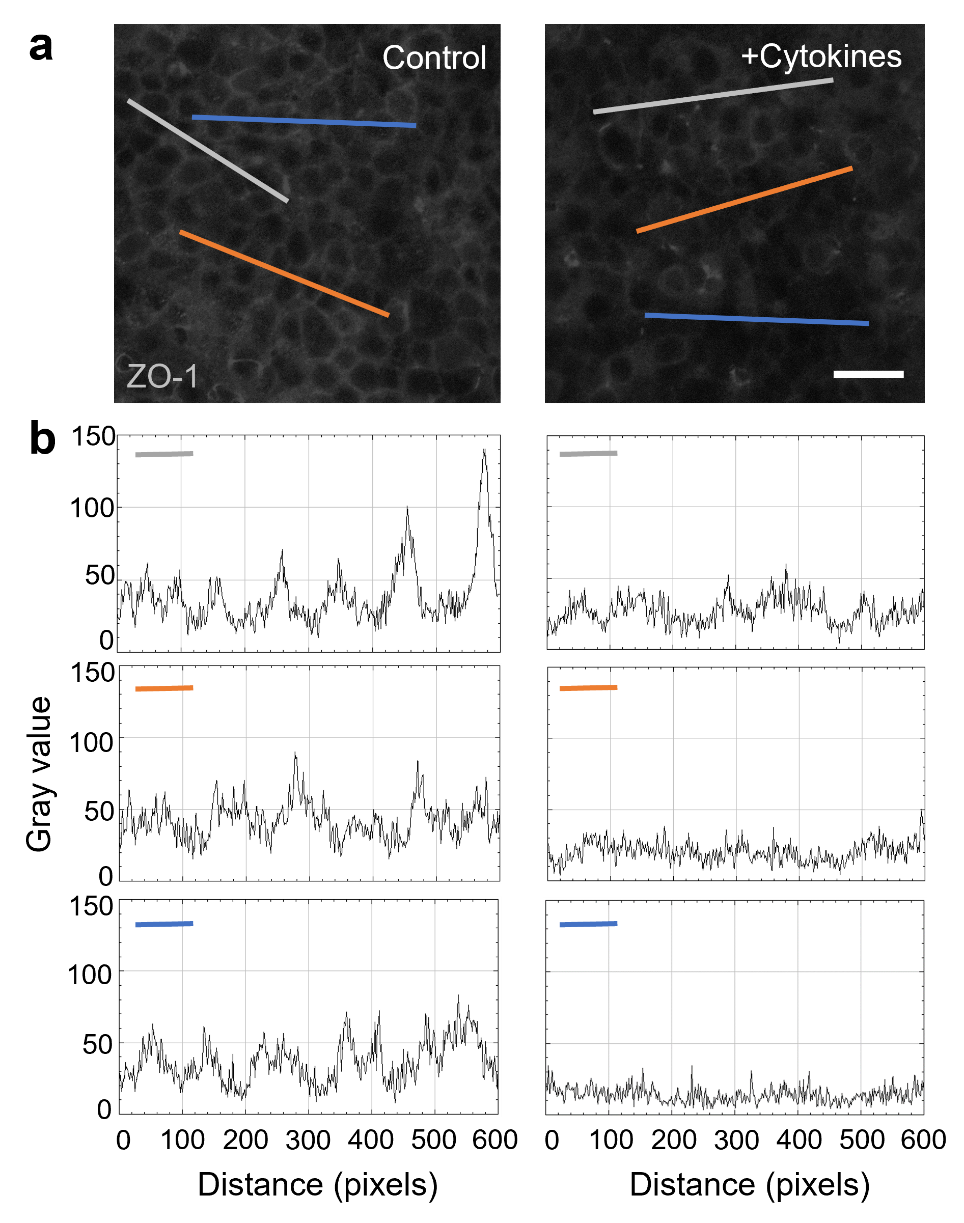
**

**Supplementary Figure S1.** Intensity profiles of fluorescent images of the epithelial cells that highlight tight junction protein, ZO-1. (**a**) The location of three independent positions (grey, blue, and orange bars) for the comparative line scan of the corresponding images provided in Fig. 2c. (**b**) The intensity profile of immunofluorescence micrographs highlighting ZO-1 in control (Control) and the cytokine-challenged groups (+Cytokines). Bar, 50 µm.


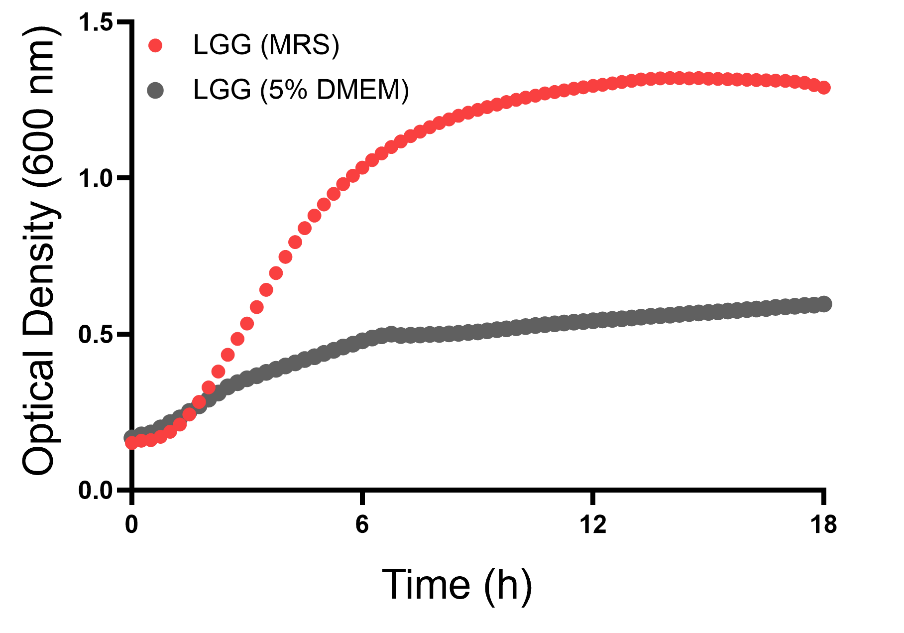


**Supplementary Figure S2.** The growth profile of LGG cells in either a bacterial culture medium (MRS) or a cell culture medium that contains 5% FBS (5% DMEM). LGG cells were grown on a shaking 96-well plate incubated in a plate reader at 37^o^C. Each culture medium was supplemented with L-cysteine (final concentration at 0.5 mg/mL; n=4). An optical density (OD) was measured at 600 nm every 15 min for 18 h.


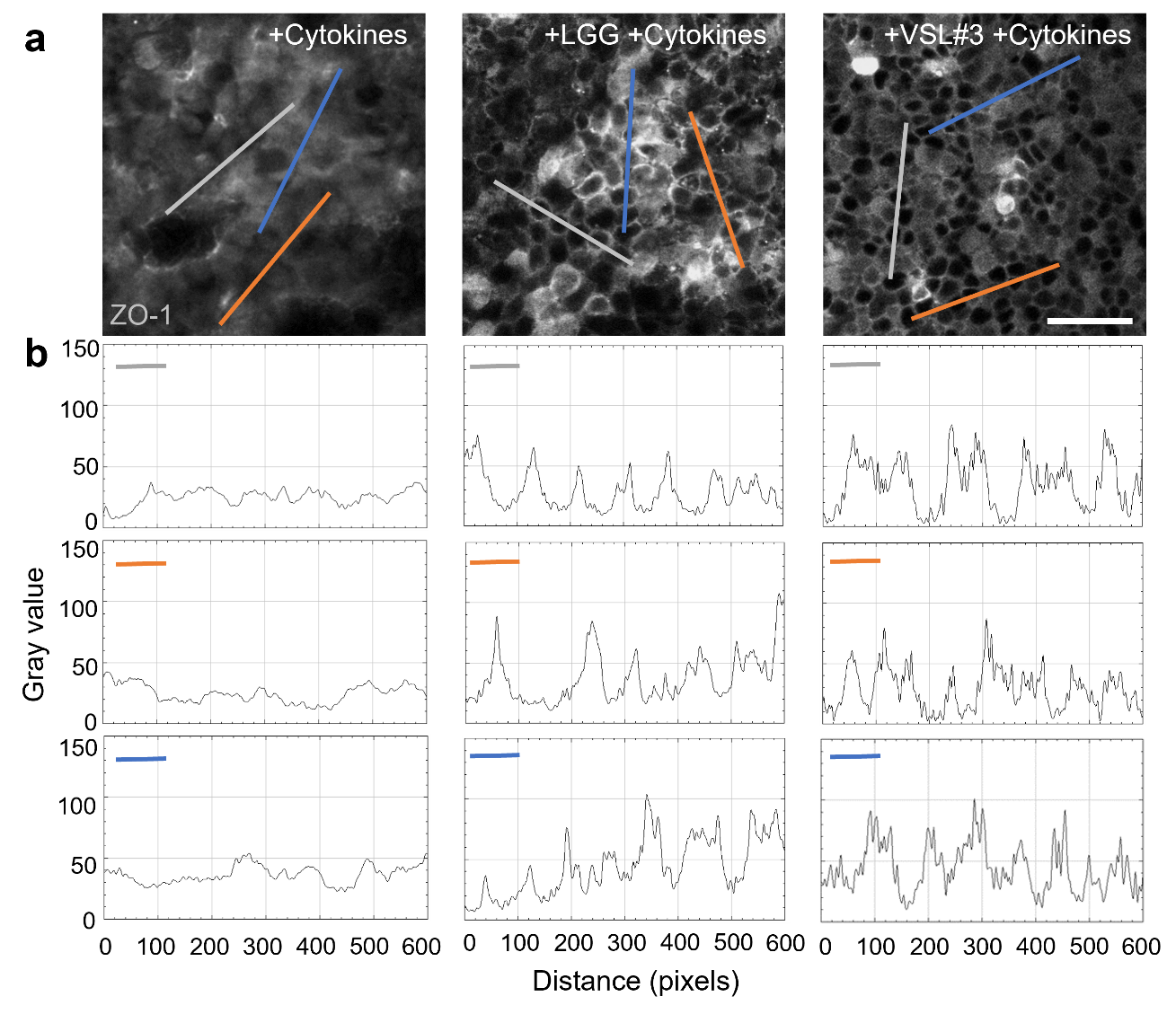


**Supplementary Figure S3.** Intensity profiles of fluorescent images of the epithelial cells that highlight tight junction protein, ZO-1. (**a**) The location of three independent positions (grey, blue, and orange bars) for the comparative line scan of the corresponding images provided in Fig. 4c “ZO-1” panel. (**b**) The intensity profile of immunofluorescence micrographs highlighting ZO-1 in the cytokine-challenged group (+Cytokines) versus the group co-cultured with LGG (+LGG +Cytokines) or VSL#3 (+VSL#3 +Cytokines). Bar, 50 µm.


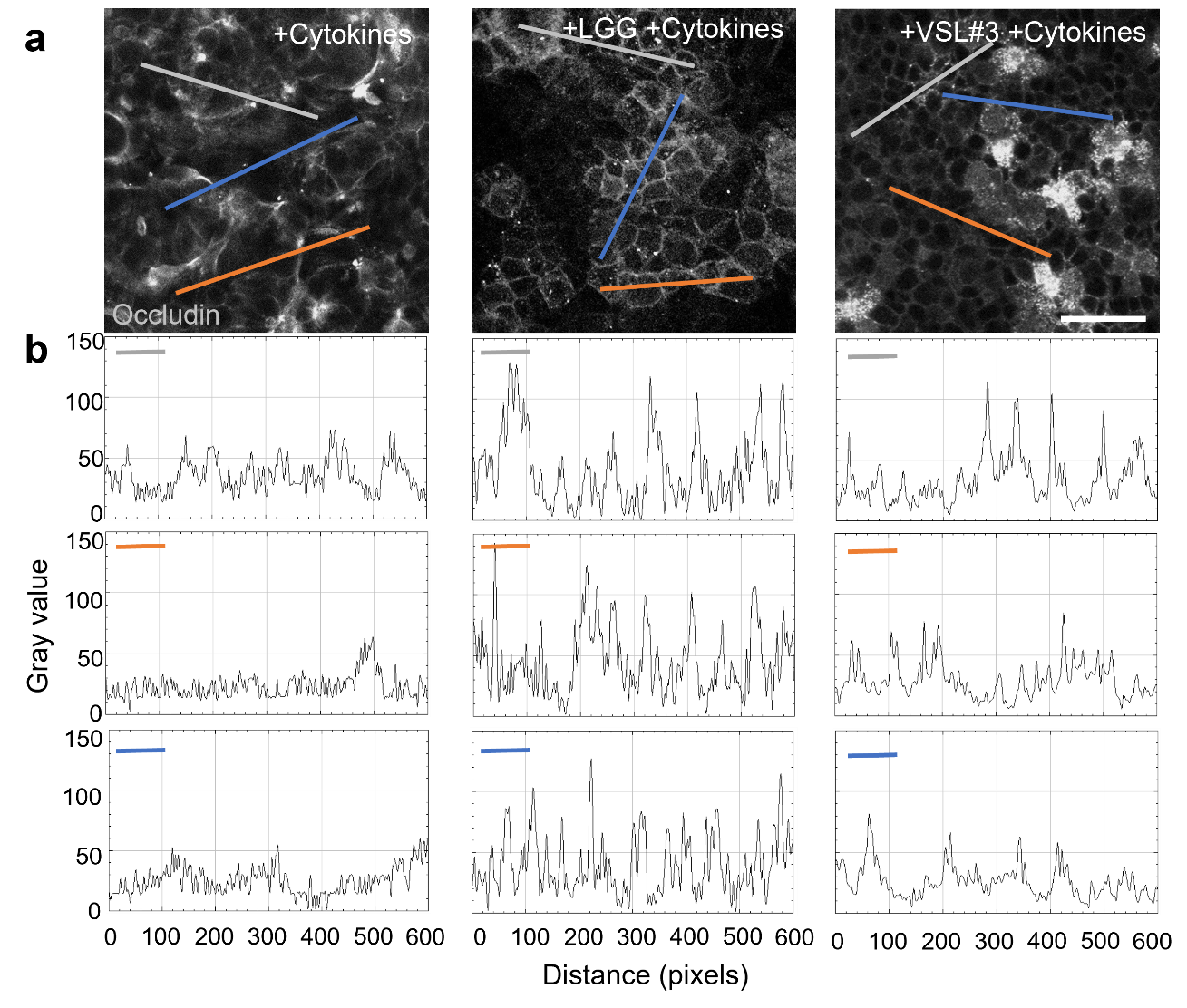


**Supplementary Figure S4.** Intensity profiles of fluorescent images of the epithelial cells that highlight tight junction protein, occludin. (**a**) The location of three independent positions (grey, blue, and orange bars) for the comparative line scan of the corresponding images provided in Fig. 4c “Occludin” panel. (**b**) The intensity profile of immunofluorescence micrographs highlighting occludin in the cytokine-challenged group (+Cytokines) versus the group co-cultured with LGG (+LGG +Cytokines) or VSL#3 (+VSL#3 +Cytokines). Bar, 50 µm.

**Supplementary Video S1.** A real-time live recording of LGG bacterial cells colonized on the apical surface of the epithelium taken on Day 3 of the co-culture in a Leaky Gut Chip.

**Supplementary Video S2.** A real-time live recording of VSL#3 bacterial cells colonized on the apical surface of the epithelium taken on Day 3 of the co-culture in a Leaky Gut Chip.
